# Supplementary material for: Reduced nitrogenase efficiency dominates response of the globally important nitrogen fixer Trichodesmium to ocean acidification
Source: Nat Commun. 2019 Apr 3;10:1521. doi: 10.1038/s41467-019-09554-7 (PMC6447586; doi:10.1038/s41467-019-09554-7)
Supplement: Supplementary file 1 — Supplementary Information [file 41467_2019_9554_MOESM1_ESM.pdf]

Supplementary Information for

Reduced nitrogenase efficiency dominates response of the globally important nitrogen fixer *Trichodesmium* to ocean acidification

Luo et al.

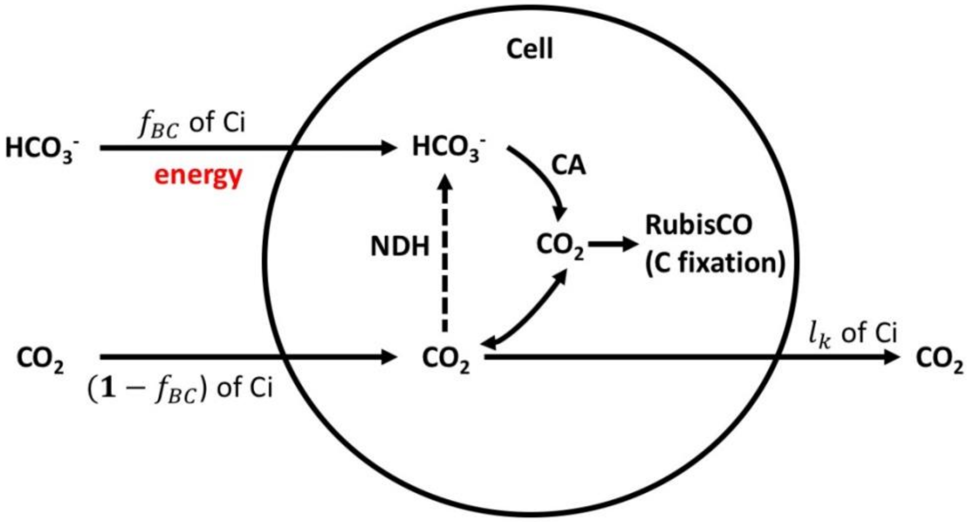

Supplementary Figure 1. A simple scheme for *Trichodesmium* CO_2-concentration mechanism (CCM). HCO_3^- transport contributes f_{BC} portion of total inorganic carbon uptake (Ci) and CO_2 contributes the rest. A portion of CO_2 is leaked out of cell in amount of l_k of Ci.

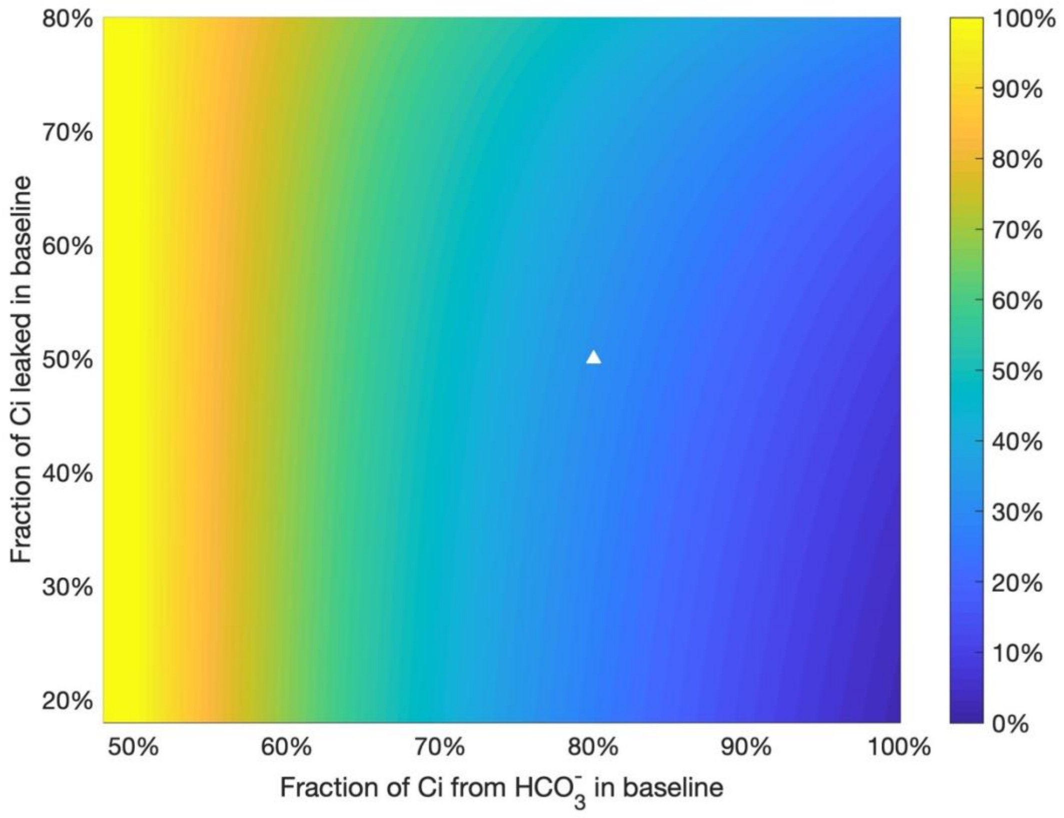

Supplementary Figure 2. Evaluation of the saving of CO_2-concentrating mechanism (CCM) energy consumption under OA. Saving of the CCM energy consumption by doubling dissolved CO_2 concentration in seawater is calculated as a function of initial fraction of total dissolved carbon uptake (Ci) contributed by HCO_3^- transport and initial fraction of Ci leaked out of cell in baseline condition. The white triangle represents the value used in this study.

(a)

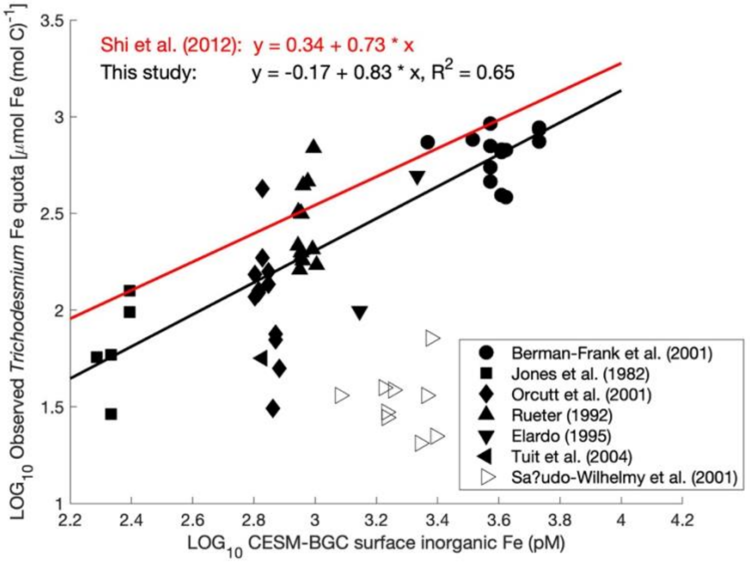

(b)

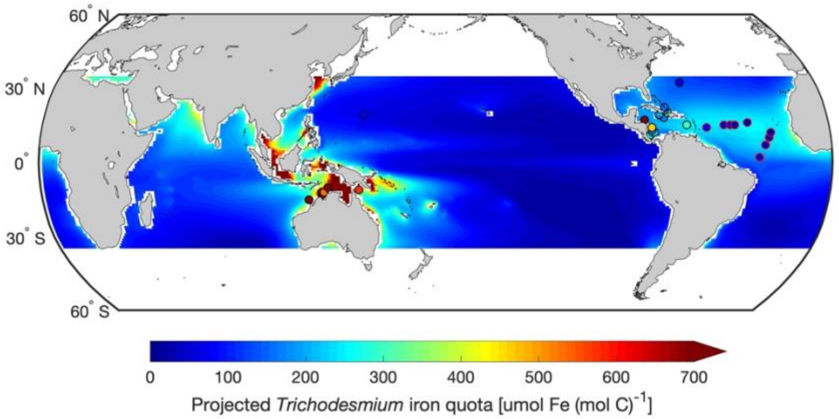

Supplementary Figure 3. The relationship of *Trichodesmium* Fe quota and seawater inorganic Fe concentration. (a) The regression (black line) of the logarithms of observed *Trichodesmium* Fe quota data [1-7] to the logarithms of modeled (CESM-BGC) surface inorganic Fe in 1990s. A dataset [6] is excluded from the regression (see Materials and Methods). Also shown is the regression line (red) of the two variables using another experimental dataset in which both variables are measured [8]. **(b)** The estimated *Trichodesmium* Fe quota from the CESM-BGC surface inorganic Fe in 1990s using the regression of this study, overlaying the measurements of *Trichodesmium* Fe quota (circles).

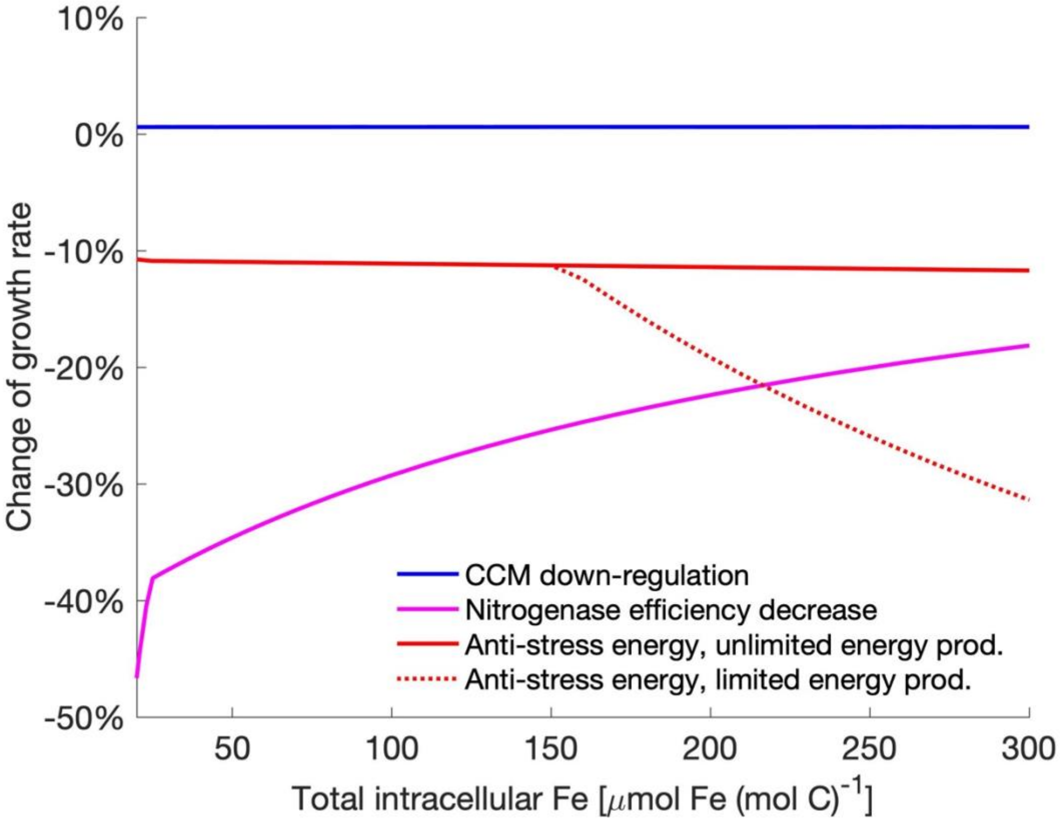

Supplementary Figure 4. Impact of anti-stress energy demand on the model results when light is limiting. The change of *Trichodesmium* growth rate with pH changed from 8.02 to 7.82 is modeled when the anti-stress energy demand is enabled as the only OA impact under two assumptions: (1) the energy production increases infinitely with photosystem Fe (solid red line), and (2) if maximum energy production rate is set to 560 kJ $(mol\ C)^{-1}\ d^{-1}$ (dashed red line). For comparison, also shown the modeled change of *Trichodesmium* growth rate when only CCM down-regulation (blue solid line) or nitrogenase efficiency decrease (magenta) is enabled as the only OA impact. Note that the solid and the dashed red lines overlap at low intracellular Fe level.

(a)

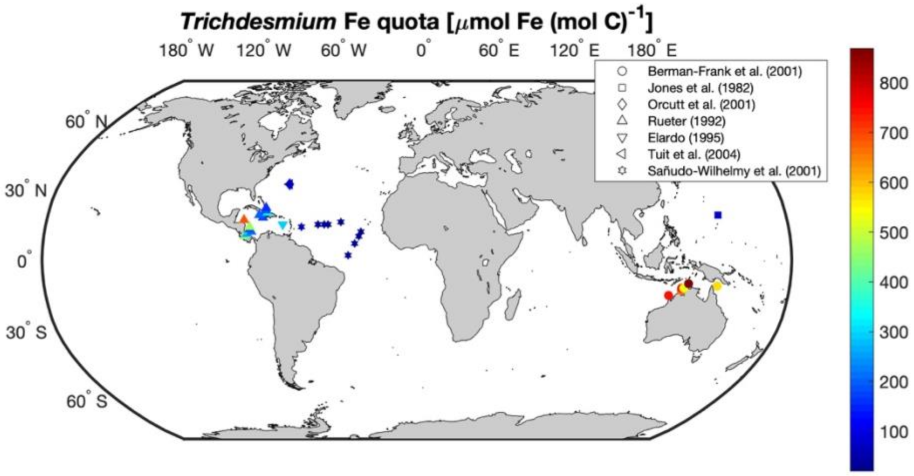

(b)

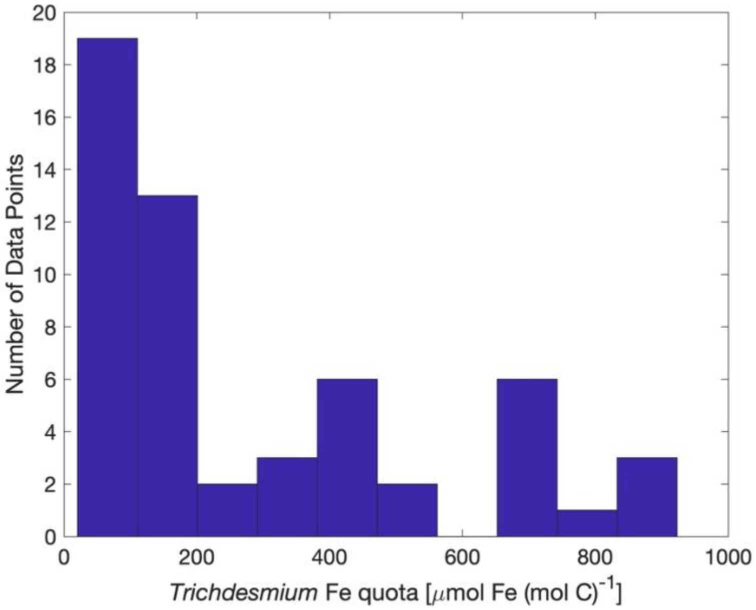

Supplementary Figure 5. Historical measurements of *Trichodesmium* intracellular Fe quota. Data were obtained from literature [1-7]. **(a)** The spatial distribution and **(b)** the histogram.

(a)

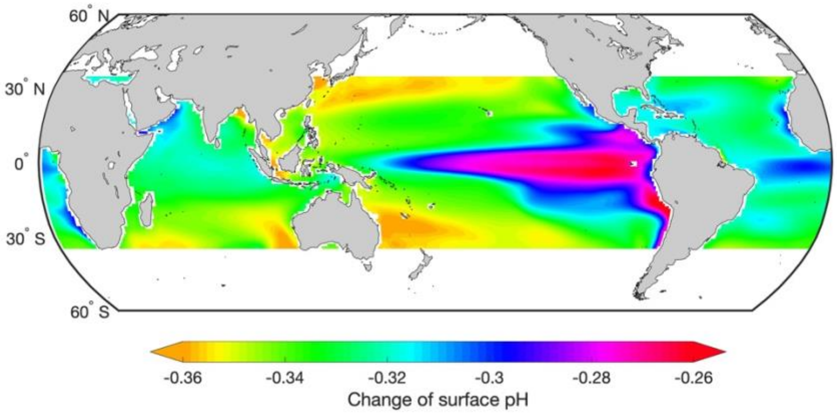

(b)

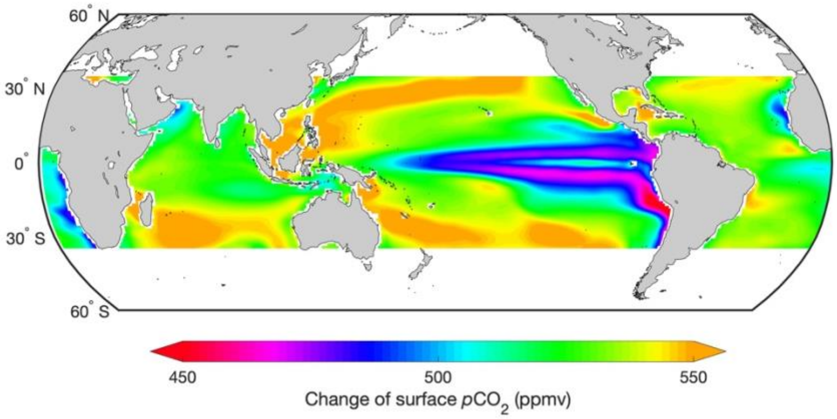

Supplementary Figure 6. Change of the CESM-BGC model outputs. Change of decadal average surface **(a)** pH and **(b)** pCO_2 from 1990s to 2090s under scenario RCP 8.5.

Supplementary Table 1. Unique spectrums of PsaC, Dps, and ferritin in *Trichodesmium*.

Protein Name	Unique Spectrum number*	Subunit ratio of protein to PsaC	Number of subunits per protein molecule	Molecular ratio of protein to PsaC
PsaC	35.6±1.2	-	1	-
Dps	28.3±1.5	0.79	12	0.066
Ferritin	106.7±16.2	3.00	24	0.125

*Data are from the same proteomic analysis as reported by Hong et al.[9]. Peptide identification at the 95% confidence interval was counted and filtered by 1% false discovery rate (FDR). Mean ± 1 s.d. (n=3) of samples from four treatments [i.e., ambient and acidified conditions at the lowest (~35 pM Fe′) and the highest (~925 pM Fe′) Fe levels, see Table 1 for details].

Supplementary Table 2. Estimation of the maximal Fe storage capacity of Dps and ferritin in *Trichodesmium*.

Protein name	Number of Fe per protein	Molecular ratio of protein to PsaC*	Ambient condition Fe quota [µmol Fe (mol C)$^{-1}$]	Acidified condition Fe quota [µmol Fe (mol C)$^{-1}$]
PsaC	12	-	8.41	11.03
Dps	260[10]	0.066	12.0	15.8
Ferritin	4,500[11]	0.125	394	517
Total storage Fe†			406	533

See Supplementary Note 1 for the calculation.

*Based on estimate of unique spectrums (Supplementary Table 1).

†Total Fe storage capacity of Dps plus ferritin.

Supplementary Table 3. Estimation of intracellular Fe allocated to nitrogenase and photosystems of *Trichodesmium* from protein content.

	# of Fe atoms per protein	Protein content [μmol (g total protein)$^{-1}$]		Fe content [μmol Fe (g total protein)$^{-1}$]		Fe quota§ [μmol Fe (mol C)$^{-1}$]	
		Ambient*	Acidified*	Ambient*	Acidified*	Ambient*	Acidified*
Low Fe (Fe′ ≈ 35 pM)							
NifH (nitrogenase)	9.5	0.235±0.028	0.394±0.075	2.23±0.27	3.74±0.71	**16.1±1.9**	**27.0±5.1**
PsbA (PSII)	3	0.064±0.011	0.090±0.024	0.19±0.03	0.27±0.07	1.39±0.23	1.94±0.52
PetC (Cyt b_6f)	6	0.023±0.003	0.009±0.002	0.14±0.02	0.05±0.01	1.01±0.11	0.39±0.09
PsaC (PSI)	12	0.036±0.006	0.014±0.004	0.44±0.08	0.16±0.04	3.13±0.55	1.18±0.32
Ferredoxin†	2	0.036±0.006	0.014±0.004	0.07±0.01	0.03±0.01	0.52±0.09	0.20±0.05
Fe in photosystems‡						**6.1±0.6**	**3.7±0.6**
High Fe (Fe′ ≈ 925 pM)							
NifH (nitrogenase)	9.5	0.552±0.067	0.638±0.050	5.24±0.64	6.06±0.48	**37.8±4.5**	**43.6±3.4**
PsbA (PSII)	3	0.083±0.010	0.084±0.005	0.25±0.03	0.25±0.02	1.79±0.21	1.82±0.11
PetC (Cyt b_6f)	6	0.033±0.000	0.047±0.008	0.20±0.00	0.28±0.05	1.42±0.02	2.04±0.34
PsaC (PSI)	12	0.097±0.001	0.128±0.009	1.17±0.01	1.53±0.11	8.41±0.09	11.03±0.76
Ferredoxin†	2	0.097±0.001	0.128±0.009	0.20±0.00	0.26±0.02	1.40±0.02	1.84±0.13
Fe in photosystems‡						**13.0±0.2**	**16.7±0.8**

Errors denote 1 SD (n = 3).

*Experiments were conducted at ambient (pCO_2≈400 μatm, pH 8.02) and acidified (pCO_2≈700 μatm, pH 7.82) conditions.

†Ferredoxin was not measured but estimated by assuming ferredoxin:PSI = 1:1.

§Fe quota was estimated by assuming proteins accounting for 30% of dry cell mass and C for 50% of dry cell mass, thus Fe content is converted to Fe quota using a conversion factor of 0.6 g protein (g C)$^{-1}$, i.e., 7.2 g protein (mol C)$^{-1}$.

‡Fe in photosystems was estimated as sum of PSII, Cyt b_6f, PSI and Ferrerdoxin.

Supplementary Note 1

Estimation for *Trichodesmium* Fe storage capacity

As antibodies and standards for Dps and ferritin (frt) are not commercially available for absolute protein quantification, we estimated their amounts based on the numbers of unique spectra obtained by mass spectrometry, assuming that the spectra numbers are proportional to the protein/peptide amounts [12,13]. PsaC as has been quantified by western blot was used as a reference. The Dps and frt subunits were roughly 79% and 3 times of PsaC, respectively (Supplementary Table 1). As each Dps protein contains 12 subunits and each frt contains 24 subunits, the abundance of Dps and frt were 6.7% and 12.5% of PsaC, respectively (Supplementary Table 1). Given each Dps protein can bind up to 260 Fe atoms [10] and each frt can bind up to 4500 Fe [11], using the measured Fe quota in PsaC (Supplementary Table 3), maximum Fe quota in Dps and frt can be calculated as:

$$Q_{\mathrm{Fe}}^{\mathrm{Dps}} = Q_{\mathrm{Fe}}^{\mathrm{PsaC}} \cdot \frac{n_{\mathrm{Fe}}^{\mathrm{Dps}}}{n_{\mathrm{Fe}}^{\mathrm{PsaC}}} \cdot r_{\mathrm{PsaC}}^{\mathrm{Dps}},$$

$$Q_{\mathrm{Fe}}^{\mathrm{frt}} = Q_{\mathrm{Fe}}^{\mathrm{PsaC}} \cdot \frac{n_{\mathrm{Fe}}^{\mathrm{frt}}}{n_{\mathrm{Fe}}^{\mathrm{PsaC}}} \cdot r_{\mathrm{PsaC}}^{\mathrm{frt}},$$

where $Q_{\mathrm{Fe}}^{\mathrm{Dps}}$ and $Q_{\mathrm{Fe}}^{\mathrm{PsaC}}$ are maximum Fe quotas in Dps and PsaC, respectively, $n_{\mathrm{Fe}}^{\mathrm{PsaC}}$ = 12 is the number of Fe atoms per PsaC, $n_{\mathrm{Fe}}^{\mathrm{Dps}}$ = 260 is the maximum number of Fe atoms per Dps, $n_{\mathrm{Fe}}^{\mathrm{frt}}$ = 4500 is the maximum number of Fe atoms per frt, and $r_{\mathrm{PsaC}}^{\mathrm{Dps}}$ and $r_{\mathrm{PsaC}}^{\mathrm{frt}}$ are ratios of Dps and frt abundance to PsaC abundance, respectively, based on the spectrum analysis. The results are listed in Supplementary Table 2.

Supplementary References

1 Berman-Frank, I., Cullen, J. T., Shaked, Y., Sherrell, R. M. & Falkowski, P. G. Iron availability, cellular iron quotas, and nitrogen fixation in *Trichodesmium*. *Limnology and Oceanography* **46**, 1249-1260 (2001).

2 Jones, G., Burdon-Jones, C. & Thomas, F. Influence of *Trichodesmium* red tides on trace metal cycling at a coastal station in the Great Barrier Reef lagoon. *Oceanologica Acta, Special issue*, 319-326 (1982).

3 Orcutt, K. M. *et al.* A seasonal study of the significance of N_2 fixation by *Trichodesmium* spp. at the Bermuda Atlantic Time-series Study (BATS) site. *Deep-Sea Research II* **48**, 1583-1608 (2001).

4 Rueter, J. G., Hutchins, D. A., Smith, R. W. & Unsworth, N. L. Iron nutrition of *Trichodesmium*, in *Marine pelagic cyanobacteria: Trichodesmium and other diazotrophs*, (eds E.J. Carpenter, D.G. Capone, & J.G. Rueter) 289-306 (Kluwer Academic Publishers, Dordrecht, 1992).

5 Elardo, K. M. *Changes in proteins associated with nitrogen fixation and iron nutrition in the marine cyanobacterium Trichodesmium* M.S. thesis, Portland State University, Portland, Oregon, (1995).

6 Sañudo-Wilhelmy, S. A. *et al.* Phosphorus limitation of nitrogen fixation by *Trichodesmium* in the central Atlantic Ocean. *Nature* **411**, 66-69 (2001).

7 Tuit, C., Waterbury, J. & Ravizzaz, G. Diel variation of molybdenum and iron in marine diazotrophic cyanobacteria. *Limnology and Oceanography* **49**, 978-990 (2004).

8 Shi, D., Kranz, S. A., Kim, J.-M. & Morel, F. M. M. Ocean acidification slows nitrogen fixation and growth in the dominant diazotroph *Trichodesmium* under low-iron conditions. *Proceedings of the National Academy of Sciences* **109**, E3094-E3100 (2012).

9 Hong, H. *et al.* The complex effects of ocean acidification on the prominent N_2-fixing cyanobacterium *Trichodesmium*. *Science* **356**, 527-531 (2017).

10 Castruita, M. *et al.* Overexpression and characterization of an iron storage and DNA-binding Dps protein from *Trichodesmium erythraeum*. *Appl. Environ. Microbiol.* **72**, 2918-2924 (2006).

11 Harrison, P. M. & Arosio, P. The ferritins: molecular properties, iron storage function and cellular regulation. *Biochim. Biophys. Acta* **1275**, 161-203 (1996).

12 Zhang, S. F. *et al.* iTRAQ-based quantitative proteomic analysis of a toxigenic dinoflagellate Alexandrium catenella and its non-toxic mutant. *Proteomics* **15**, 4041-4050 (2015).

13 Lundgren, D. H., Hwang, S.-I., Wu, L. & Han, D. K. Role of spectral counting in quantitative proteomics. *Expert Review of Proteomics* **7**, 39-53 (2010).
